# Verification of serum albumin elevating effect of cell-free and concentrated ascites reinfusion therapy for ascites patients: a retrospective controlled cohort study

**DOI:** 10.1038/s41598-019-46774-9

**Published:** 2019-07-15

**Authors:** Yosuke Yamada, Keita Inui, Yuuta Hara, Kazuaki Fuji, Kosuke Sonoda, Koji Hashimoto, Yuji Kamijo

**Affiliations:** Department of Nephrology, Shinshu University School of Medicine, 3-1-1 Asahi, Matsumoto, Nagano 390-8621 Japan

**Keywords:** Medical research, Cancer therapy

## Abstract

Cell-free and concentrated ascites reinfusion therapy (CART) is frequently used to treat refractory ascites in Japan. However, its efficacy remains unclear. This controlled cohort study verified the serum albumin elevating effect of CART by comparisons with simple paracentesis. Ascites patients receiving CART (N = 88) or paracentesis (N = 108) at our hospital were assessed for the primary outcome of change in serum albumin level within 3 days before and after treatment. A significantly larger volume of ascites was drained in the CART group. The change in serum albumin level was +0.08 ± 0.25 g/dL in the CART group and −0.10 ± 0.30 g/dL in the paracentesis group (P < 0.001). The CART – paracentesis difference was +0.26 g/dL (95%CI +0.18 to +0.33, P < 0.001) after adjusting for potential confounders by multivariate analysis. The adjusted difference increased with drainage volume. In the CART group, serum total protein, dietary intake, and urine volume were significantly increased, while hemoglobin and body weight was significantly decreased, versus paracentesis. More frequent adverse events, particularly fever, were recorded for CART, although the period until re-drainage was significantly longer. This study is the first demonstrating that CART can significantly increase serum albumin level as compared with simple paracentesis. CART represents a useful strategy to manage patients requiring ascites drainage.

## Introduction

Refractory ascites patients often develop hypoalbuminemia due reasons such as protein leakage into ascites^1^ and body protein consumption by underlying diseases^2,3^. Since albumin is essential for the maintenance of colloid osmotic pressure, hypoalbuminemia can cause systemic swelling, intravascular dehydration, and prerenal failure^4–6^. Albumin also plays important roles in the upkeep and transportation of drugs and other intravascular substances. Hence, hypoalbuminemia can reduce the medical efficacy of diuretics and anti-cancer agents^7,8^. Many epidemiology studies have demonstrated hypoalbuminemia as an independent poor prognosis factor in various diseases^9–11^. In liver cirrhosis and malignant tumors, which are the main causes of ascites, hypoalbuminemia was found to be an important risk factor associated with lifetime and infection-treatment prognosis^12–16^. Therefore, hypoalbuminemia is a serious problem for ascites patients not only for a worsened prognosis, but also for diminished quality of life by systemic swelling, drug resistance, and other conditions. Treatment and prevention methods for hypoalbuminemia are critically important.

Ascites drainage by paracentesis is performed as a general non-drug treatment for refractory ascites^17,18^. During simple paracentesis, hypoalbuminemia may develop since large amounts of protein are drained and discarded^1^. To prevent hypoalbuminemia by the loss of ascites protein^19^, cell-free and concentrated ascites reinfusion therapy (CART) was developed in Japan in the 1970s^20^. After ascites drainage in the CART procedure, cancer and other cells along with bacteria are eliminated by a filter membrane and the protein in the remaining ascites is concentrated by removing excess water with a concentrator membrane. The final product is then reinfused into the patient’s vein (Supplementary Video S1)^21^. CART is frequently performed in Japan^22^ and has undergone several improvements, such as establishing the safety of draining large amounts of ascites^23–28^, indication expansion to cancerous ascites^29,30^, and the development of external pressure type filtration methods whose filter membrane can be easily washed^24,25^. However, CART is relatively obscure outside of Japan, possibly since its efficacy remains unclear. As almost all studies on CART are case series with no set controls, evaluating the clinical merits of CART is difficult^31^. Even controlled comparisons with simple paracentesis, which is the most common non-drug treatment for ascites^17,18^, have not been conducted^32^. The objective of the current investigation was to verify the serum albumin elevating effect of CART by controlled study with simple paracentesis. We conducted a retrospective cohort study using medical records at our hospital to compare the changes in serum albumin level before and after treatment between CART and paracentesis.

## Methods

### Study design, setting, and participants

This was a single-center, retrospective, controlled cohort study.

The medical records of ascites patients receiving CART or paracentesis during hospitalization at Shinshu University Hospital between June 2011 and June 2017 were extracted. During the studied period, 310 CART sessions and 477 paracentesis sessions (total: 787 drainage treatment sessions; herewith, CART and paracentesis are collectively termed “drainage treatment”) were performed (Supplementary Fig. S1). Since many patients received 2 or more drainage treatments, the number of individual patients who received CART or paracentesis was counted. In the case that a patient received both CART and simple paracentesis, the data for CART and paracentesis were treated as 2 different patients. A total of 107 patients receiving CART (number of sessions: 2.9 ± 3.0 per patient) and 177 patients undergoing paracentesis (number of sessions: 2.7 ± 2.7 per patient) remained after this step.

### Eligibility criteria

Based on following eligibility criteria, sessions whose data were used for analysis were selected from patient medical records. The inclusion criteria were: (1) patient age of at least 20 years at the drainage treatment, (2) more than 500 mL of ascites was drained, and (3) serum albumin level was measured within 3 days before and after drainage treatment. The exclusion criteria were: (1) bacterial peritonitis at the time of drainage treatment, and (2) 2 or more drainage treatments carried out between pre- and post-treatment albumin measurement.

To prevent bias towards patients receiving numerous treatments, 1 session per patient was used for analysis. When a patient received 2 or more drainage treatments, the data of the first session were used if it fulfilled the eligibility criteria. If not, the second and subsequent sessions were considered in chronological order. Patients without an eligible session were excluded.

Ultimately among the 107 CART and 177 paracentesis patients, the number of eligible sessions was 88 in the CART group and 108 in the paracentesis group (overlap cases: 42). In the CART group, data from the first session was used in 85% (75) of cases, followed next by the second session in 11% (10), third session in 2% (2), and fifth session in 1% (1). In paracentesis group, data from the first session was used in 81% (88) of cases, followed next by the second session in 14% (15) and third session in 5% (5).

### Exposure and control

**Figure 1:**
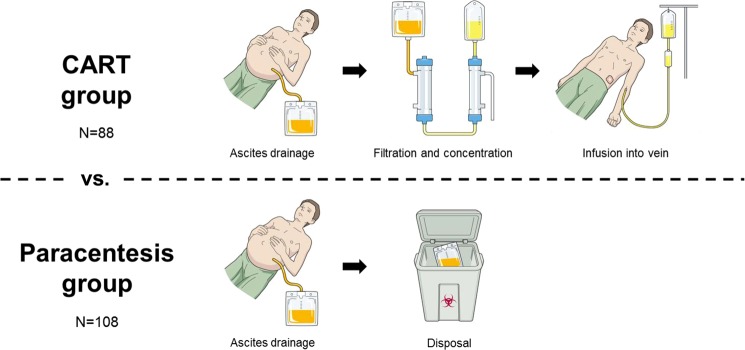
Conceptual diagram of this study (CART vs. paracentesis). CART group: after ascites drainage, cancer and other cells and bacteria in the ascitic fluid are eliminated with a filter membrane and excess water is removed with a concentrator membrane. The final product is infused back into the patient’s vein. Paracentesis group: after drainage, all ascites is discarded. The copyright holder, Yosuke Yamada, permits to publish these images under a CC BY open access license.

The 88 sessions of ascites patients receiving CART (exposure) were compared with the 108 sessions of simple paracentesis (control) (Fig. 1). All CART treatments in this study employed low and external pressure filtration (DC-CART)^24^ with a filter membrane washing feature to clear membrane clogging and to prevent premature termination of ascites processing. AHF-MOW (Asahi Kasei Medical Co., Tokyo, Japan) and AHF-UP (Asahi Kasei Medical Co.) were used as the filter membrane and concentrator membrane, respectively.

### Collection of baseline characteristics of studied patients and processing conditions

The following patient parameters were collected: age and sex at the time of drainage treatment, body height at the time of hospitalization, body weight within 3 days before drainage treatment, body temperature and mean blood pressure determined by a nurse in the morning of the day of drainage treatment, dietary intake from breakfast consumption data on the day prior to drainage treatment as observed and recorded by nurses, and daily urine volume on the day prior to drainage treatment. Regarding comorbidities, the existence of cancer, liver cirrhosis, and infectious disease requiring antibiotics was recorded. Laboratory data were obtained from blood samples obtained within 3 days before drainage treatment as well as from the drained ascites. Estimated glomerular filtration rate (eGFR) was calculated using an equation established for the Japanese: eGFR = 194 × Serum creatinine^−1.094^ × Age^−0.287^ (×0.739 if female)^33^. As combined treatment, data on transfusion, diuretics, and a sodium-restricted diet were also collected. The total amounts of albumin preparation (ALBP), fresh frozen plasma (FFP), and red cell concentrate (RCC) that were transfused intravenously between pre- and post-treatment blood testing were counted. ALBP amount was determined as the weight of albumin contained in the albumin preparation in consideration of the difference in albumin concentrations among different kinds of ALBP. The use of diuretics classified as “C03 diuretics” by the Anatomical Therapeutic Chemical Classification System (high-ceiling diuretics, low-ceiling diuretics, carbonic anhydrase inhibitors, potassium-sparing agents, and vasopressin antagonists) were recorded^34^. Patients following a sodium-restricted diet (salt <7 g) were counted as well^31^. Fever as a side effect of CART has been reported, and fever-prevention medication, such as steroids and non-steroidal anti-inflammatory agents (NSAIDs), is sometimes used just prior to CART^22,24^. Therefore, in the CART group, the use and frequency of fever-prevention drugs were also investigated.

Processing conditions were recorded for each eligible drainage treatment session. Concentration ratio was calculated as: Volume of drained ascites (mL)/Volume of reinfused ascites (mL). Protein collection efficiency was determined as: (Concentration of total protein in reinfused ascites [g/dL] × Volume of reinfused ascites [dL])/(Concentration of total protein in drained ascites [g/dL] × Volume of drained ascites [dL]).

### Primary outcome

The short-term Δ serum albumin level was determined as: Albumin level within 3 days after treatment – Albumin level within 3 days before treatment. For all parameters, “Δ” denotes the change between before and after drainage treatment. Since serum albumin level was measured within 3 days before and after drainage treatment, measurement timing deviated slightly among subjects. However, serum albumin levels were not expected to change greatly due to a long biological half-life of approximately 25 days^35^. If 2 or more blood tests were conducted within the 3 days before or after drainage treatment, the data closer to the treatment day were used.

### Secondary outcomes

For long-term Δ serum albumin level, Δ serum albumin level at 1 week after drainage treatment was calculated as: Value at 1 week after treatment – Value before treatment. Δ serum albumin level at 2 weeks after drainage treatment was determined as: Value at 2 weeks after treatment – Value before treatment. Serum albumin levels measured between 4 and 10 days and between 11 and 17 days after treatment were collected as the values of 1 and 2 weeks after drainage treatment, respectively. For values before treatment, the above-described serum albumin level within 3 days before drainage treatment was used.

The Δ other blood data included Δ serum total protein, Δ eGFR, Δ serum total bilirubin, Δ white blood cell count, Δ hemoglobin, and Δ platelet count. Blood data measured together with serum albumin level within 3 days before and after drainage treatment were used for calculations.

The Δ clinical data was assessed as Δ mean blood pressure, Δ daily urine volume of the treatment day, Δ daily urine volume of the following day, Δ dietary intake, and Δ body weight. Δ mean blood pressure was calculated as: Value in the morning on the following day of drainage treatment – Value in the morning on the drainage treatment day. Δ daily urine volume of the treatment day was determined as: Volume on the drainage treatment day – Volume on the day prior to drainage treatment. Δ daily urine volume of the following day was measured as: Volume on the following day of drainage treatment – Volume on the day prior to drainage treatment. Δ dietary intake was calculated as: Breakfast consumption on the following day of drainage treatment – Breakfast consumption on the day prior to drainage treatment. Δ body weight was calculated as: Body weight measured within 3 days after drainage treatment – Body weight measured on the day prior to drainage treatment.

Information regarding adverse events was collected from doctor and nurse records. The rate of ascites re-drainage within 60 days after drainage treatment was investigated from medical records. For both CART and paracentesis, re-drainage during either an outpatient visit or hospitalization was counted.

### Sample size estimation

Based on the results of post-marketing CART surveillance reported by Hanafusa *et al*.^22^, Δ serum albumin level with a difference between the 2 groups of 0.4 g/dL and a standard deviation (SD) of 0.75 g/dL were estimated. The exposure group:control group ratio was set as 2:3 from a clinical perspective. Therefore, a sample size of 155 cases (62 CART and 93 paracentesis) was calculated to provide 90% power to detect differences at a significance level of 0.05 (2-sided). Approximately 40% of the initial dataset was estimated to miss the eligibility criteria. Accordingly, 284 patients (107 CART and 177 paracentesis) were included in this study. Power and Sample Size Calculation software version 3.1.2 (Vanderbilt University, Nashville, TN, USA) was used for sample size determinations.

### Statistical analysis

For each analysis, a 2-sided significance level of P = 0.05 was used. Quantitative data were expressed as the mean ± SD. Qualitative data were expressed as the percentage (number). Each parameter was compared between the CART and paracentesis groups. In univariate analysis, the Student *t*-test for quantitative data, the chi-square test for qualitative data, and the log-rank test for survival analysis were employed. In multivariate analysis adjusting for potential confounders, multiple linear regression analysis for quantitative data and Cox proportional hazards regression analysis for survival analysis were used. For analysis of the primary outcome, stratified comparisons by ascites drainage volume were performed as sensitivity analysis (model 3 in multivariate analysis). Missing data were not included in analyses. Calculations were performed by means of the IBM SPSS Statistics software package version 20 for Windows (IBM Co., Ltd., New York, NY, USA).

### Ethics approval and consent to participate

This study was performed in accordance with the tenets set forth in the Declaration of Helsinki and approved by the ethics committee of Shinshu University Hospital (authorization number: 3904). Informed written consent was waived in this study due to its retrospective nature using medical records that did not subject the patients to new interventions. The collected data were anonymously stored and used for analysis. Furthermore, as an alternative to written informed consent, an opt-out document was created and posted on the hospital website that contained information on the design of the research and publication of the results to provide subjects the opportunity to halt the provision of their medical data. None of the patients refused to provide data.

## Results

### Patient characteristics and processing conditions of each treatment

Among patient characteristics, age, sex, physique, body temperature, urine volume, and dietary intake did not differ remarkably between the CART group and paracentesis group, although mean blood pressure was significantly higher for paracentesis (P = 0.032) (Table 1). The comorbidity rates of ascites-producing diseases, such as cancer and liver cirrhosis, did not differ greatly between the groups, while the prevalence of infectious diseases apart from bacterial peritonitis, which was excluded by eligibility criteria, was significantly higher in paracentesis patients (P = 0.010). The collected blood and ascites laboratory data before drainage treatment were comparable for CART and paracentesis. Regarding combined blood transfusion therapy, the usage rates of ALBP and FFP were significantly higher in the paracentesis group (P < 0.001 and P = 0.027, respectively). In contrast, the rate of RCC use tended to be higher for CART (P = 0.068). The use of diuretics did not differ between the groups (P = 0.664). The ratio of a sodium-restricted diet was 40% (35) in the CART group and 34% (37) in the paracentesis group, which were comparable (P = 0.617). In the CART group, 32% (28) had been pretreated with steroids for fever prevention. No patients had used NSAIDs for fever prophylaxis.

**Table 1:** Characteristics of studied patients and combined transfusion therapy.

Characteristic	CART (N = 88)	Paracentesis (N = 108)	P-value
Age (years)	62 ± 12	61 ± 12	0.390
Male	41% (36)	38% (41)	0.670
Body height (cm)	158.8 ± 7.7	159.1 ± 8.7	0.850
Body weight (kg)	58.3 ± 12.3	59.9 ± 12.4	0.380
Body temperature (°C)	36.7 ± 0.4	36.8 ± 0.5	0.069
Mean blood pressure (mmHg)	83.9 ± 12.1	88.0 ± 13.7	0.032
Daily urine volume (mL/day)	616 ± 497	696 ± 430	0.320
Dietary intake (%)	29 ± 34	25 ± 34	0.400
***Comorbidities***			
All cancer	90% (79)	87% (94)	0.550
Ovarian cancer^†^	19% (17)	26% (28)	0.270
Liver cell cancer^†^	11% (10)	11% (12)	0.960
Uterine cancer^†^	11% (10)	7% (8)	0.340
Liver cirrhosis	18% (16)	19% (21)	0.820
Infectious diseases	15% (13)	30% (32)	0.010
***Laboratory data***			
Serum total protein (g/dL)	5.9 ± 0.8	5.9 ± 0.8	0.630
Serum albumin (g/dL)	2.4 ± 0.5	2.5 ± 0.5	0.480
eGFR (mL/min/1.73 m^2^)	59 ± 31	62 ± 28	0.490
Serum total bilirubin (mg/dL)	2.1 ± 4.1	3.1 ± 5.7	0.230
C-reactive protein (mg/dL)	5.5 ± 5.5	6.7 ± 5.8	0.155
White blood cells (/μL)	7,830 ± 5,404	9,210 ± 6,965	0.140
Hemoglobin (g/dL)	9.8 ± 1.8	10.2 ± 2.2	0.080
Platelets (×10^4^/μL)	27.2 ± 17.2	27.7 ± 18.5	0.840
Ascitic total protein (g/dL)	2.8 ± 1.5	2.9 ± 1.7	0.760
Ascitic albumin (g/dL)	1.4 ± 0.8	1.5 ± 0.9	0.290
***Combined therapy***			
Use of ALBP	13% (11)	38% (41)	<0.001
Amount of ALBP in using patients (g)	18.2 ± 6.5	18.9 ± 9.7	
Use of FFP	2% (2)	10% (11)	0.027
Amount of FFP in using patients (units)	3.0 ± 1.4	3.5 ± 2.4	
Use of RCC	10% (9)	4% (4)	0.068
Amount of RCC in using patients (units)	2.2 ± 0.7	2.0 ± 0.0	
Use of diuretics	43% (38)	40% (43)	0.664

^†^Top 3 types among all cancers in this cohort. Qualitative data, percentage (number); P-values were calculated by the chi-square test. Quantitative data, mean ± SD; P-values were calculated by the Student *t*-test.

Concerning the processing conditions of each treatment, the volume of drained ascites was significantly larger in the CART group (P < 0.001) (Table 2). The concentration ratio and protein collection efficiency of CART were 11.9 ± 10.7 and 59 ± 23%, respectively. All of the drained ascites underwent filtration and concentration, with no premature termination of the procedure.

**Table 2:** Processing conditions of CART and paracentesis.

	CART^†^ (N = 88)	Paracentesis (N = 108)	P-value
Volume of drained ascites (mL)	4,159 ± 2,570	2,662 ± 1,590	<0.001
Volume of reinfused ascites (mL)	591 ± 446	—	—
Concentration ratio	11.9 ± 10.7	—	—
Concentration of total protein in reinfused ascites (g/dL)	11.1 ± 3.1	—	—
Concentration of albumin in reinfused ascites (g/dL)	6.1 ± 1.9	—	—
Weight of total protein in reinfused ascites (g)	67.7 ± 51.2	—	—
Weight of albumin in reinfused ascites (g)	36.4 ± 27.6	—	—
Protein collection efficiency (%)	59 ± 23	—	—
Premature termination	0% (0)	—	—

^†^Low and external pressure type filtration method CART (DC-CART^23^). Qualitative data, percentage (number). Quantitative data, mean ± SD; P-value was calculated by the Student *t*-test.

### Δ serum albumin level

**Figure 2:**
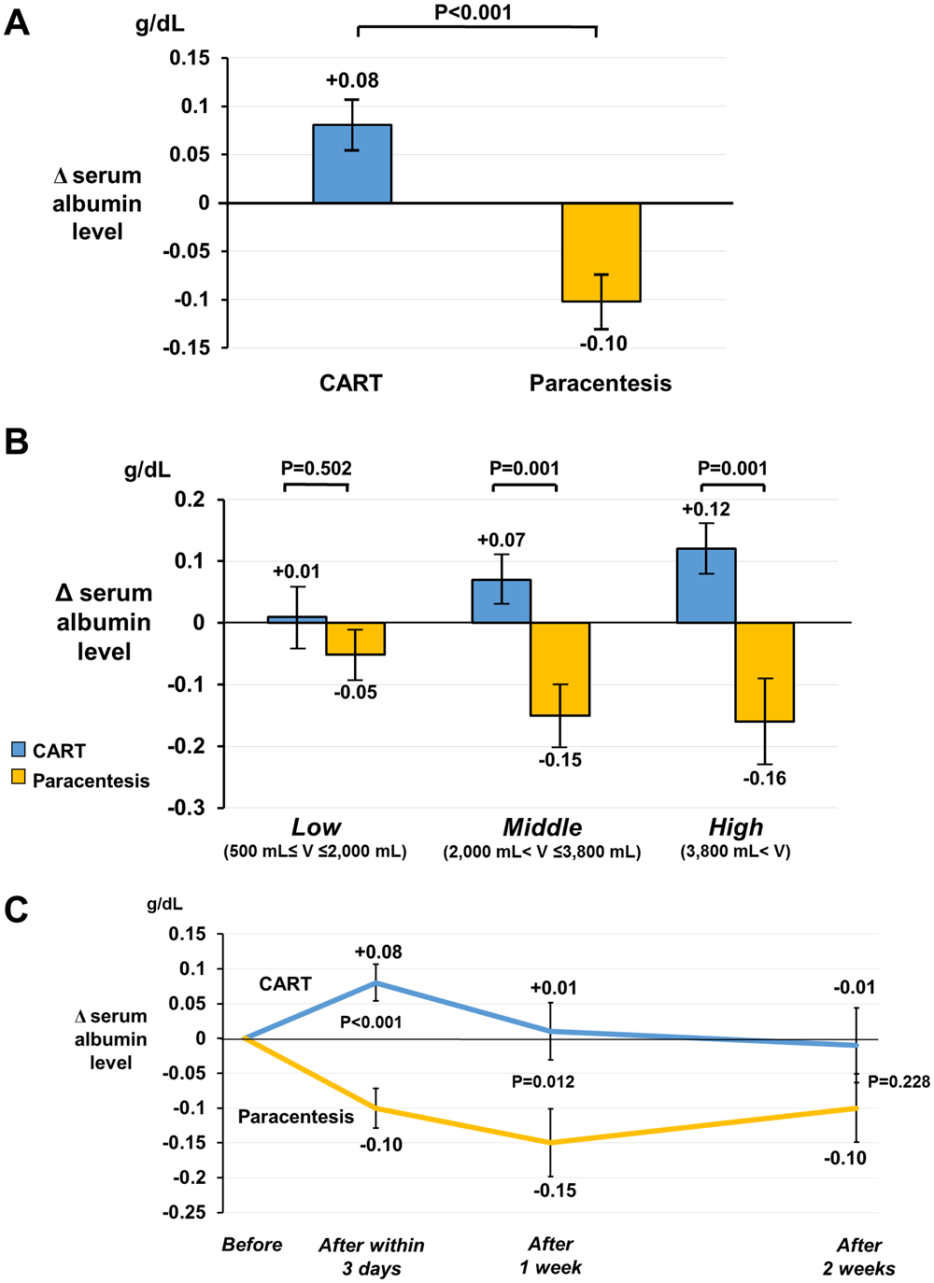
Δ serum albumin level in the CART and paracentesis groups. (**A**) Short-term Δ serum albumin level was compared between the CART group (N = 88) and the paracentesis group (N = 108) by univariate analysis (Student *t*-test). (**B**) Drained ascites volume was divided into tertiles (low volume layer, 11 CART and 56 paracentesis; middle volume layer, 41 CART and 27 paracentesis; high volume layer, 36 CART and 25 paracentesis) for stratified analysis. Univariate analysis by the Student *t*-test was employed. (**C**) Long-term Δ serum albumin level following drainage treatment. Comparisons between the groups were performed using the Student *t*-test. The number of data samples used for the analysis at 1 week after drainage treatment was 73 CART and 90 paracentesis, while that at 2 weeks was 60 CART and 83 paracentesis. Blue bar, CART group; yellow bar, paracentesis group; blue line, CART group; yellow line, paracentesis group. Error bars represent standard error. Abbreviation: V, volume.

The primary outcome of short-term Δ serum albumin level was compared between the groups. In univariate analysis, Δ serum albumin level was significantly higher in the CART group (+0.08 ± 0.25 g/dL vs. −0.10 ± 0.30 g/dL, P < 0.001) (Fig. 2A). Figure 2B presents the analysis of drained ascites volume divided into tertiles (low, 500 mL ≤ volume ≤ 2,000 [33.3 percentile] mL; middle, 2,000 mL < volume ≤ 3,800 [66.6 percentile] mL; high, 3,800 mL < volume). Although Δ serum albumin level was not remarkably different in the low volume layer (CART: +0.01 ± 0.29 g/dL vs. paracentesis: −0.05 ± 0.16 g/dL, P = 0.502), those of the CART group for the middle and high volume layers were significantly higher (middle volume layer, CART: +0.07 ± 0.25 g/dL vs. paracentesis: −0.15 ± 0.24 g/dL, P = 0.001; high volume layer, CART: +0.12 ± 0.27 g/dL vs. paracentesis: −0.16 ± 0.36 g/dL, P = 0.001). Moreover, the Δ serum albumin level of CART gradually increased with the volume of drained ascites, while that of the paracentesis group decreased. Since those results were influenced by potential confounders, we performed multivariate analysis to adjust for them (Table 3). Based on the characteristics of this cohort and clinical perspectives, we judged the 3 blood transfusion therapies (i.e., amounts of ALBP, FFP, and RCC) and comorbid infectious diseases as confounders^36,37^ and selected them as adjustment factors. In model 1, the difference in Δ serum albumin level (CART – paracentesis) was not adjusted for any confounders. In model 2, the difference was adjusted for the above 4 confounders. In model 3, the difference was adjusted for the above 4 confounders as well as ascites drainage volume by stratified analysis. In all models, Δ serum albumin level was significantly higher in the CART group as compared with the paracentesis group. In model 3, the adjusted difference became larger as the drained ascites volume increased. A sensitivity analysis was also performed using the data from the 42 overlap cases that were treated with both CART and paracentesis in order to study a population with similar background conditions. Comparisons of short-term Δ serum albumin by this analysis supported the effects of CART (Supplementary Fig. S2).

**Table 3:** Difference in Δ serum albumin level between CART and paracentesis adjusted by multiple linear regression analysis.

Adjustment level	Adjusted difference in Δ serum albumin level (g/dL) [CART – paracentesis] (95%CI)	P-value
Model 1: Unadjusted	+0.18 (+0.11 to +0.26)	<0.001
Model 2: ALBP, FFP, RCC, infectious disease	+0.26 (+0.18 to +0.33)	<0.001
Model 3: Model 2 + stratified by ascites drainage volume		
Low (500 mL ≤ volume ≤ 2,000 mL)	+0.16 (+0.01 to +0.31)	0.036
Middle (2,000 mL < volume ≤ 3,800 mL)	+0.28 (+0.16 to +0.40)	<0.001
High (3,800 mL < volume)	+0.43 (+0.26 to +0.59)	<0.001

Δ indicates change from pre-treatment to post-treatment. Multiple linear regression analysis was used.

Figure 2C depicts the long-term Δ serum albumin level after each drainage treatment. The Δ serum albumin level at 1 week after treatment differed significantly between the groups in univariate analysis (CART: +0.01 ± 0.35 g/dL vs. paracentesis: −0.15 ± 0.46 g/dL, P = 0.012). Serum albumin levels at 2 weeks after drainage treatment in the CART group remained near pretreatment levels, although the significant difference in Δ serum albumin level between the groups disappeared (CART: −0.01 ± 0.41 g/dL vs. paracentesis: −0.10 ± 0.44 g/dL, P = 0.228). Since ALBP and FFP were considered the most important confounders, a sensitivity analysis was performed by excluding patients receiving ALBP or FFP between within 3 days and 14 days after drainage treatment. The effects of CART were also evident in this analysis (Supplementary Fig. S3), and the significant difference between the groups persisted until 2 weeks later.

### Δ other blood and clinical data

The Δ other blood data were compared in Table 4. In the CART group, Δ serum total protein level was significantly higher (P < 0.001) and Δ hemoglobin level was significantly lower (P = 0.032) than in the paracentesis group. Serum total protein level increased and hemoglobin level decreased after treatment in CART patients. Although not statistically significant, Δ total bilirubin level tended to be higher and Δ platelets count tended to be lower in the CART group (P = 0.085 and P = 0.086, respectively).

**Table 4:** Differences in secondary outcomes between CART and paracentesis.

Outcome	Univariate analysis^†^			Multivariate analysis^‡^	
	CART	Paracentesis	P-value	Adjusted difference of each outcome [CART – paracentesis] (95%CI)	P-value
Δ total protein (g/dL)	+0.1 ± 0.5	−0.2 ± 0.5	<0.001	+0.4 (+0.2 to +0.5)	<0.001
Δ eGFR (mL/min/1.73 m^2^)	+2.4 ± 12.1	+1.6 ± 11.2	0.627	+1.3 (−2.2 to +4.9)	0.461
Δ total bilirubin (mg/dL)	+0.2 ± 0.8	−0.01 ± 0.5	0.032	+0.2 (−0.03 to +0.4)	0.085
Δ white blood cell (/μL)	−438 ± 2,752	+249 ± 2,667	0.086	−673 (−1,517 to +171)	0.117
Δ hemoglobin (g/dL)	−0.6 ± 1.2	−0.3 ± 0.8	0.023	−0.3 (−0.6 to −0.03)	0.032
Δ platelets (×10^4^/µL)	−2.2 ± 6.0	−0.8 ± 5.2	0.098	−1.5 (−3.3 to +0.2)	0.086
Δ mean blood pressure (mmHg)	−6.0 ± 11.8	−8.8 ± 18.4	0.233	+3.1 (−1.7 to +7.9)	0.206
Δ daily urine volume of the treatment day (mL/day)	+350 ± 659	+114 ± 437	0.029	+253 (+42 to +464)	0.019
Δ daily urine volume of the next day (mL/day)	+266 ± 525	+119 ± 474	0.114	+178 (−21 to +377)	0.079
Δ dietary intake (%)	+16 ± 30	+5 ± 33	0.022	+12 (+1 to +22)	0.029
Δ body weight (kg)	−3.8 ± 2.7	−2.0 ± 0.2	<0.001	−1.7 (−2.5 to −0.9)	<0.001

Δ indicates change from pre-treatment to post-treatment. ^†^Indication method, mean ± SD; P-values were calculated by the Student *t*-test.

^‡^Multiple linear regression analysis was used. Difference of each outcome was adjusted for ALBP, FFP, RCC, and infectious diseases.

Regarding the Δ clinical data, Δ daily urine volume of the treatment day and Δ dietary intake were significantly higher in the CART group (P = 0.019 and P = 0.029, respectively). Daily urine volume and dietary intake increased after treatment in both groups. Body weight was lower postoperatively in both groups, but more significantly in the CART group (P < 0.001).

### Adverse events

The number of patients who developed any adverse event was significantly higher in the CART group over the paracentesis group (P < 0.001) (Table 5). The incidence of fever was particularly high among CART patients (P < 0.001). No severe adverse events were recorded for either group. Stratification analysis by drainage volume showed that anemia tended to more frequently occur with high volumes in the CART group (Supplementary Table S1).

**Table 5:** Adverse events.

Event	CART (N = 88)	Paracentesis (N = 108)	P-value
All patients with any adverse event	25% (22)	6% (6)	<0.001
Fever^†^	15% (13)	2% (2)	<0.001
Anemia^‡^	6% (5)	1% (1)	0.092
Pain around puncture site	2% (2)	2% (2)	1.000
Chest pain	1% (1)	1% (1)	1.000
Vomiting	1% (1)	0% (0)	0.450
Chills	1% (1)	0% (0)	0.450
Wobble	1% (1)	0% (0)	0.450
Hematoma around puncture site	1% (1)	0% (0)	0.450

There were no fatal adverse events. ^†^Defined as body temperature elevated by over 1 °C and reaching over 38 °C. ^‡^Defined as hemoglobin decreased by over 1 g/dL and reaching less than under 7 g/dL. Indication method, percentage (number); P-values were calculated by the chi-square test.

### Rate of ascites re-drainage within 60 days after treatment

**Figure 3:**
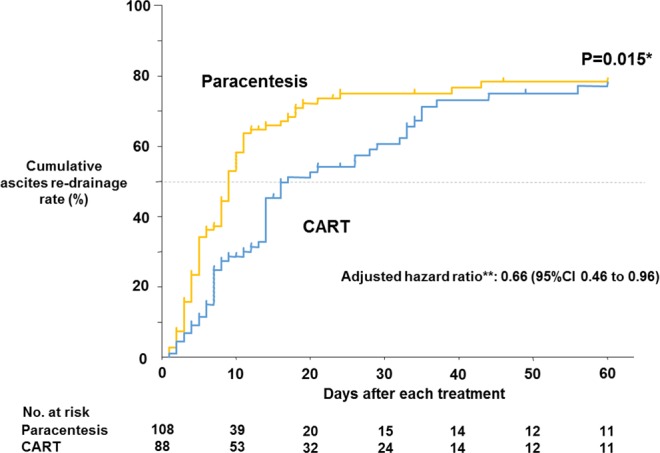
Kaplan-Meier comparison of ascites re-drainage rate within 60 days after drainage treatment. *Ascites re-drainage rate within 60 days after treatment was compared between the 2 groups by the log-rank test. **Adjusted hazard ratio was calculated by Cox proportional hazards regression analysis corrected for ALBP, FFP, RCC, and infectious diseases. Blue line, CART group; yellow line, paracentesis group.

The duration until re-drainage was significantly longer in the CART than in the paracentesis group (P = 0.015) (Fig. 3). Re-drainage was performed in 55 (CART 69% [38], paracentesis 31% [17]) of 88 cases in the CART group during the observation period. Re-drainage in the paracentesis group was done in 75 (CART 21% [16], paracentesis 79% [59]) of 108 cases. The ascites re-drainage rate for CART was 3.0 cases per 100 person-days, while that for paracentesis was 4.6 cases per 100 person-days (Adjusted hazard ratio: 0.66 [95%CI +0.46 to +0.96], P = 0.023). The cumulative ascites re-drainage rate reached 50% at 17 days in the CART group and 9 days in the paracentesis group. Thus, the number of days to reach 50% was 8 days longer in CART patients.

## Discussion

We investigated the efficacy of CART in this controlled cohort study by comparisons with simple paracentesis to clearly demonstrate a serum albumin elevating effect. Other advantages of CART over paracentesis included increased drainage volume, serum total protein, dietary intake, and urine volume as well as a longer period until re-drainage.

The number of controlled studies on CART is very small, with only 4 randomized controlled trials (RCTs) to date^38–41^. Moreover, the controls in all 4 of the RCTs were not simple paracentesis, but rather paracentesis with ALBP infusion for ethical and other considerations (Supplementary Table S2). In those papers, precise comparisons between CART and paracentesis were considered impossible because the ALBP infusion accompanying paracentesis also imparted serum albumin elevating effects (Supplementary Fig. S4A). As a result, the CART group did not show a serum albumin elevating effect over the paracentesis group modified by ALBP infusion in any of the 4 studies. Also in our study, there was no significant difference in albumin elevating effect when comparing the 77 subjects in the CART alone group with the 41 patients in the paracentesis plus ALBP infusion group (Supplementary Fig. S5). The majority of other studies on CART have been case series without controls, whereby serum albumin level was simply compared between before and after treatment. However, during CART, it is thought that ascites drainage by paracentesis decreases serum albumin level, which is then restored by concentrated ascites reinfusion. Therefore, the difference between serum albumin before and after CART is difficult to recognize (Supplementary Fig. S4B). Albumin level changes prior to and following CART are also affected by other factors, such as combined blood transfusion therapy and comorbidities. In the current study, ethical issues were avoided by adopting a retrospective design. A significant serum albumin elevating effect of concentrated ascites reinfusion could be demonstrated by comparing CART with simple paracentesis using multivariate analysis adjusted for potential confounders, including combined blood transfusion (Supplementary Fig. S4C). Furthermore, the difference in Δ serum albumin level between the groups persisted until 1 week after treatment, indicating that ascites albumin infused intravenously can remain in the circulation for an extended period. CART may thus prevent systemic edema, intravascular dehydration, and prerenal failure in the long term to reduce medical expenses for blood transfusion therapy.

Another reason why the current study of CART vs. simple paracentesis is important is that it clearly shows the effects of CART in the real clinical setting. Comparisons of CART alone vs. paracentesis with ALBP infusion are scientifically interesting in that they examine the difference between the effects of ALBP infusion and concentrated ascites infusion. However, since the combined use of CART and ALBP infusion is permitted in the clinical setting and the two are often used together, the analysis of “only CART alone can be used” as an intervention group may create applicability problems in the real world (Supplementary Fig. S6). Moreover, in the 4 RCTs of CART vs. paracentesis with ALBP infusion, the ALBP dose in the control group was arbitrarily determined by the researchers of each study and differed among the papers (Supplementary Table S2). The difference detected in the studies may therefore have been different from the clinical effects of CART observed by the medical care workers and patients. In the present study, a simple and perhaps more realistic comparison of CART vs. simple paracentesis could be performed by adjusting for the influences of ALBP and other confounders.

For CART, the adjusted difference in Δ serum albumin level (CART - paracentesis) as well as Δ serum albumin level itself for CART increased along with drainage ascites volume. These results suggested a time-course change in serum albumin level (Supplementary Fig. S7). At first, serum albumin level decreases by ascites drainage dependently on drained volume. Then, the intravenous infusion of ascitic albumin provides a serum albumin elevating effect larger than the albumin loss with drained volume to ultimately raise serum albumin level. Hence, serum albumin level appears to increase during CART more readily as the drained volume becomes larger.

Since the main aim of CART is to maintain serum albumin level^19^, it can be said that the utility of the technique increases with drainage volume. However, when draining and processing large amounts of ascites, there is a risk of filter membrane clogging with cells and proteins, resulting in premature termination of the filtration process. In such cases, the remaining original ascites is often discarded, which decreases the amount of available ascites product and negates the advantages of CART. Thus, an effective washing process for the filter membrane is needed^24^. Several methods of CART have been developed^22,24,25,42^. Among them, variations with an easy method for filter membrane washing, such as the external pressure filtration type, appear favorable^24^. Indeed, the DC-CART used in the current study successfully managed all ascites volumes in this series.

A greater amount of ascites was drained in the CART group than in the paracentesis group in our cohort. When massive ascites (i.e., 4 to 6 liters) is drained by simple paracentesis, paracentesis-induced circulatory dysfunction (PICD), such as kidney dysfunction and blood pressure drop that is associated with mortality, is reported to occur in approximately 80% of patients^43^. To prevent PICD, it is recommended to limit drainage volume or combine ALBP infusion for massive amounts of ascites^43–46^. Since ALBP infusion therapy is expensive^47^ and may increase the risk of transfusion-related infections^48–50^, physicians tend to limit drainage volume in simple paracentesis. On the other hand, multiple reports have demonstrated the safety of removing large amounts of ascites in CART^23–28^. Operators can therefore increase ascites volumes towards complete drainage of the abdominal cavity^22,24,25^. The significant difference in drained ascites between CART and simple paracentesis may have been due to the greater safety of CART in that no patient developed shock or severe adverse events in spite of nearly double the drainage volume.

Other favorable effects were observed in the CART group as compared with the paracentesis group, including higher serum total protein. During CART, proteins other than albumin, such as immunoglobulins, are also reinfused, which may help humoral immunity^51^. Dietary intake also improved more in the CART group to presumably enhance nutritional state. Body weight was more greatly reduced in the CART group. The reason for this was considered to be increased drainage and urine volume, which might have improved physical activity. Importantly, the duration until re-drainage was extended in CART patients, likely stemming from larger amounts of ascites being drained by CART and/or the maintenance of colloid osmotic pressure attenuating the leakage of intravenous water into the abdominal cavity^4^. This time extension can decrease the number and duration of hospital stays, improve patient quality of life by reducing fatigue due to frequent paracentesis^52^, and reduce medical expenses. However, there were several undesirable effects of CART. First, hemoglobin level decreased more in the CART group. Although there was no significant difference with the paracentesis group, platelets tended to be lower after CART as in previous reports^21^. It is thought that the infusion of concentrated ascites by CART exerts high colloid osmotic pressure and stimulates water shift from the extravascular space, which might dilute intravascular blood and reduce hemoglobin and platelet level^53^. Since the hemoglobin reduction by CART was minor, it would likely be insignificant for patients with normal hemoglobin level, but might pose a risk to patients whose hemoglobin is very low. Although the prophylactic administration of RCC is useful for preventing severe anemia, RCC may also cause such transfusion-related complications as infection. Therefore, we advise RCC transfusion before CART only for patients with severe anemia (i.e., hemoglobin level <8 g). In particular, since anemia tends to occur more frequently in CART with large amounts of ascites drainage, extra caution is required in such cases. Second, the total number of patients experiencing any adverse event was larger in the CART group, particularly for fever. In this study, roughly a third of patients in the CART group were given steroids to prevent fever, so the incidence of fever might have been higher if steroids were not used^22^. Many studies corroborate a higher frequency of fever in CART^22,24,25,54^, suggesting an association with intravascular cytokine infusion in the reinfused ascites^55^. However, almost all cases of fever abated after the procedure^22,24^, supporting the relative safety of CART. Regarding blood pressure, values dropped slightly (6 mmHg) on the day following CART, which was comparable to findings in the paracentesis group in spite of the drainage amount in the CART group being higher. No patients displayed any complications related to this small blood pressure decrease. Since Ito *et al*. reported that blood pressure decreased most between after drainage and before reinfusion of concentrated ascites during CART^21,29^, we also examined blood pressure during this period. However, blood pressure was similar to that on the day after CART (Supplementary Fig. S8). This difference from Ito’s results might have been due to variations in CART methods and measurement timing.

This investigation had several limitations. First, it was a single-center study that adopted DC-CART. Therefore, the mean concentration ratio was slightly higher and mean protein collection efficiency was slightly lower than the results in the post-marketing surveillance of CART^22^. When the current findings are extrapolated to other types of CART, this difference requires consideration. Especially at hospitals using CART devices with more difficult filter membrane washing, the efficacy demonstrated in this study might not be achievable due to clogging and premature termination. Second, the eligibility criteria excluded roughly 30% of patients, particularly those without serum albumin data within 3 days before or after treatment. Such exclusion may have generated selection bias. Third, the timing of laboratory data acquisition varied among patients as they were taken within 3 days before and after treatment. The measurement times of blood pressure and body temperature were also not strictly defined. Accordingly, the time until blood testing after treatment was 1.2 ± 0.1 days in the CART group and 1.5 ± 0.1 days in the paracentesis group, which was a significant difference (P = 0.004). With a long half-life^35^, serum albumin was unlikely affected, although short-lived substances, such as serum creatinine, may have been influenced. Fourth, this study was a retrospective observational study, and so we could not consider other unmeasured factors or outcomes. It should therefore be noted that some unmeasured and/or unpredictable confounders were also not considered in our calculations, especially for secondary outcomes. In the transition of serum albumin level after 1 week and 2 weeks in Fig. 2C, a sensitivity analysis (Supplementary Fig. S3) excluding the influence of ALBP and FFP, which were considered the most important confounders, was performed and showed virtually identical results. However, it was not possible to account for the effects of other additional treatments (primary disease treatment, infectious disease treatment, ascites re-draining treatment, etc.) that occurred in the period before the 14^th^ day after drainage treatment. Other outcomes, including abdominal circumference, detailed vital sign changes during CART, physical activity, and quality of life indicators, were also unmeasured and not available. Further studies that consider these outcomes are desirable. Fifth, although it was interesting that the CART group showed a longer period until re-drainage, it will be necessary to consider other unmeasured confounders and sources of bias. Particularly in Japan, if CART is re-performed in less than 14 days, national medical health insurance does not fully cover the treatment costs^56^. Thus, there might have been patients whose the time to re-drainage was intentionally extended or shortened for a treatment strategy considering this insurance practice rule. However, in cases of less than 14 days after CART, re-drainage is generally performed by simple paracentesis, with no influence by insurance policies. In agreement with this, 31% of the patients who received CART in our cohort received simple paracentesis as re-drainage treatment. Therefore, we assumed that the effect of this bias on outcomes was minor. Additional prospective studies that consider how to cope with the impact of insurance rules are desirable. Lastly, the sample size determined for this study was insufficient for performing detailed subgroup analysis with multivariate analysis, so we could not conduct investigation on the heterogeneity of the results. This investigation revealed various favorable effects of CART; however, it is unknown whether they are equally beneficial for all patients. Since many receiving CART have terminal-stage disease and systemic conditions can vary considerably, the adaptation and processing conditions of CART should be considered carefully on an individual basis.

In conclusion, this retrospective controlled study comparing CART and simple paracentesis in ascites patients is the first to demonstrate that concentrated ascites reinfusion can significantly elevate serum albumin level. The effects of CART were also thought to increase along with drained ascites volume. In CART, a larger amount of ascites could be drained than in paracentesis. CART also exhibited the benefits of longer duration until re-drainage and increases in urine volume and dietary intake. Taken together, CART represents an effective treatment option for ascites that merits greater consideration for dissemination worldwide.

## Supplementary information

Supplementary Figure and Table

Supplementary video 1-What is CART?

## Data Availability

All data analyzed during the current study are available from the corresponding author on reasonable request.
