# Supplementary material for: Verification of serum albumin elevating effect of cell-free and concentrated ascites reinfusion therapy for ascites patients: a retrospective controlled cohort study
Source: Sci Rep. 2019 Jul 15;9:10195. doi: 10.1038/s41598-019-46774-9 (PMC6629637; doi:10.1038/s41598-019-46774-9)
Supplement: Supplementary file 1 — Supplementary Figure and Table [file 41598_2019_46774_MOESM1_ESM.docx]

**Supplementary information**

**Verification of serum albumin elevating effect of**

**cell-free and concentrated ascites reinfusion therapy for ascites patients:**

**a retrospective controlled cohort study**

Yosuke Yamada, Keita Inui, Yuuta Hara, Kazuaki Fuji, Kosuke Sonoda, Koji Hashimoto and Yuji Kamijo*

Department of Nephrology, Shinshu University School of Medicine, 3-1-1 Asahi, Matsumoto, Nagano, 390-8621, Japan

*Address for correspondence:

Yuji Kamijo

Department of Nephrology, Shinshu University School of Medicine

3-1-1 Asahi, Matsumoto, Nagano 390-8621, Japan

Phone: +81-263-37-2634; Fax: +81-263-32-9412

E-mail: [yujibeat@shinshu-u.ac.jp](mailto:yujibeat@shinshu-u.ac.jp)

***
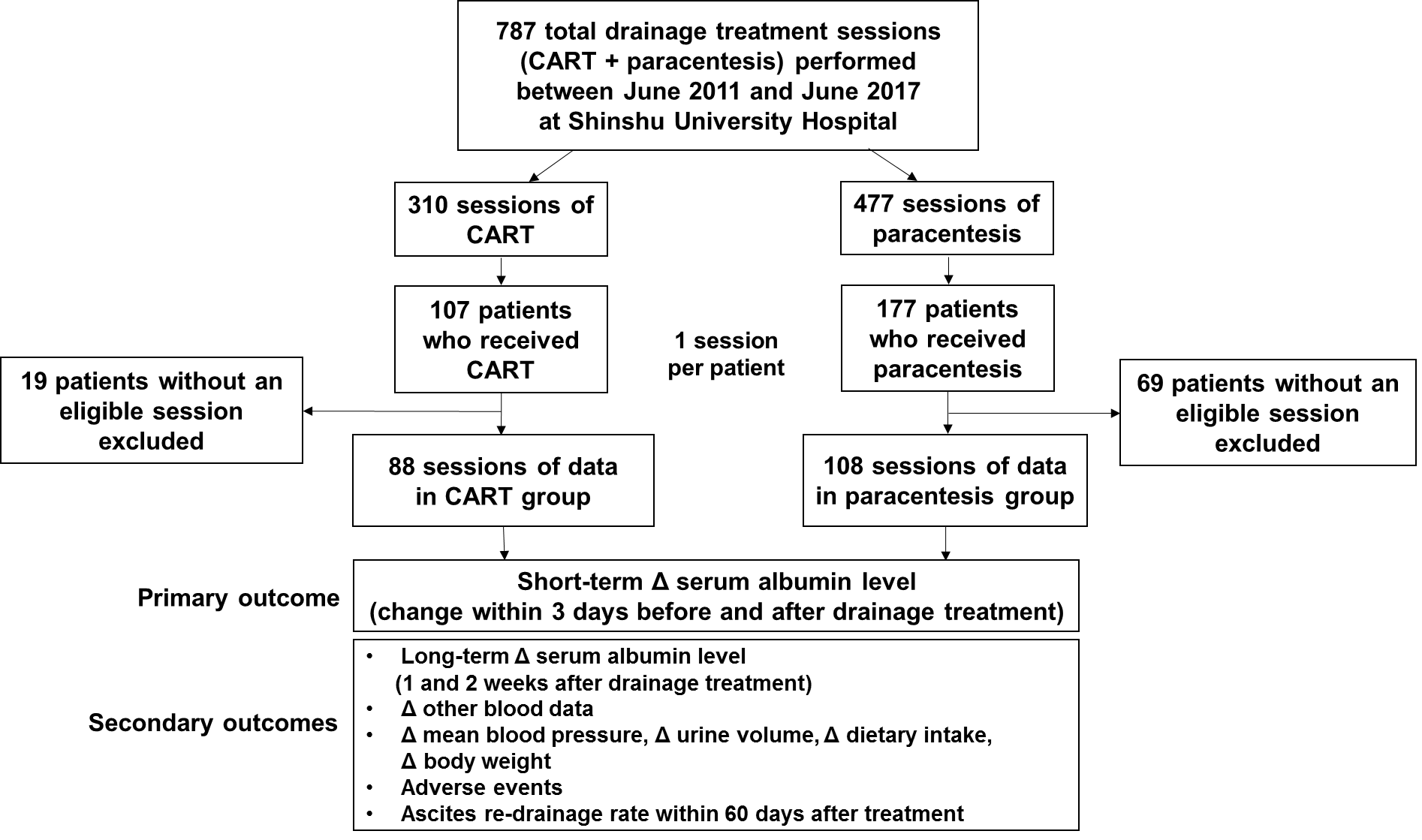
***

***Supplementary Figure S1. Patient selection data*.**


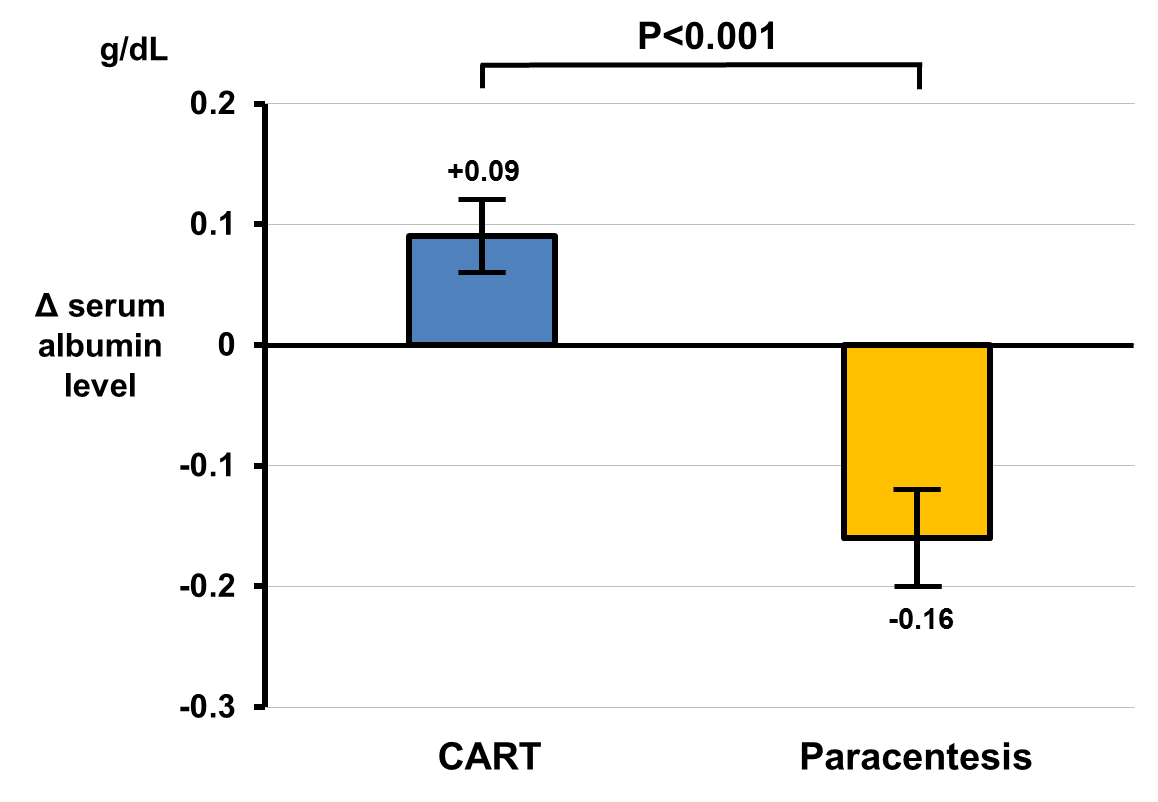


Difference in Δ serum albumin level between CART and paracentesis groups adjusted by multiple linear regression analysis

| Adjustment level | Adjusted difference in Δ serum albumin level (g/dL) [CART – paracentesis] (95%CI) |
| --- | --- |
| Model 1: Unadjusted | +0.24 (+0.13 to +0.35) |
| Model 2: ALBP, FFP, RCC, infectious disease | +0.30 (+0.19 to +0.41) |
| Model 3: Model 2 + stratified by ascites drainage volume |  |
| Low (500 mL≤ volume ≤2,000 mL) | +0.17 (-0.08 to +0.43) |
| Middle (2,000 mL< volume ≤3,800 mL) | +0.30 (+0.13 to +0.46) |
| High (3,800 mL< volume) | +0.46 (+0.17 to +0.75) |

***Supplementary Figure S2. Sensitivity analysis of overlap cases (42 CART and 42 paracentesis).*** Δ indicates change from pre-treatment to post-treatment. For statistical analysis comparing CART and paracentesis in overlap cases, the Student’s *t*-test and multiple linear regression analysis were used. Blue bar, CART; yellow bar, paracentesis; Error bars represent standard error.


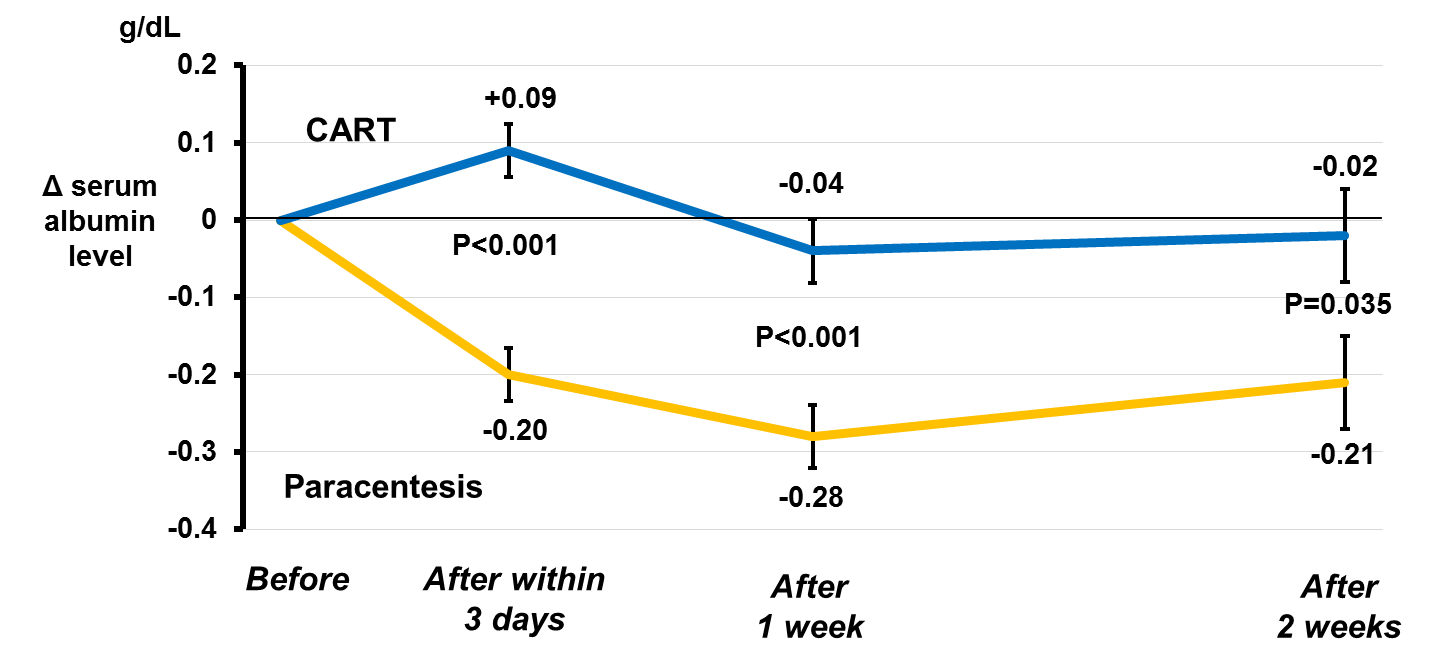


***Supplementary Figure S3. Diagram showing long-term Δ serum albumin level following drainage treatment excluding patients receiving ALBP or FFP between within 3 days and 14 days after drainage treatment.*** The number of data samples used for the analysis was as follows: After within 3 days, CART group N=56 and paracentesis group N=54; After 1 week, CART group N=56 and paracentesis group N=54; After 2 weeks, CART group N=44 and paracentesis group N=41. Comparisons between the groups were performed using the Student’s *t*-test. Blue line, CART group; yellow line, paracentesis group. Error bars represent standard error.

Supplementary Table S1. Stratified analysis of adverse events by drainage volume

| Event |  |  | Low volume  (500 mL≤ volume ≤2,000 mL) | |  | Middle volume  (2,000 mL< volume ≤3,800 mL) | |  | High volume  (3,800 mL< volume) | |
| --- | --- | --- | --- | --- | --- | --- | --- | --- | --- | --- |
|  |  |  | CART (N=11) | Paracentesis (N=56) |  | CART (N=41) | Paracentesis (N=27) |  | CART (N=36) | Paracentesis (N=25) |
| All patients with any adverse event |  |  | 9% (1) | 7% (4) |  | 22% (9) | 4% (1) |  | 33% (12) | 4% (1) |
| Fever † |  |  | 9% (1) | 2% (1) |  | 17% (7) | 0% (0) |  | 14% (5) | 4% (1) |
| Anemia ‡ |  |  | 0% (0) | 2% (1) |  | 0% (0) | 0% (0) |  | 14% (5) | 0% (0) |
| Pain around puncture site |  |  | 0% (0) | 2% (1) |  | 2% (1) | 4% (1) |  | 3% (1) | 0% (0) |
| Chest pain |  |  | 0% (0) | 2% (1) |  | 0% (0) | 0% (0) |  | 3% (1) | 0% (0) |
| Vomiting |  |  | 0% (0) | 0% (0) |  | 2% (1) | 0% (0) |  | 0% (0) | 0% (0) |
| Chills |  |  | 0% (0) | 0% (0) |  | 0% (0) | 0% (0) |  | 3% (1) | 0% (0) |
| Wobble |  |  | 0% (0) | 0% (0) |  | 0% (0) | 0% (0) |  | 3% (1) | 0% (0) |
| Hematoma around puncture site |  |  | 0% (0) | 0% (0) |  | 0% (0) | 0% (0) |  | 3% (1) | 0% (0) |

† Defined as body temperature elevated by over 1 ºC and reaching over 38 ºC. ‡ Defined as hemoglobin decreased by over 1 g/dL and reaching less than under 7 g/dL. Indication method, percentage (number)

| Supplementary Table S2. Review of previous randomized controlled studies considering the effects of CART | | | | | |
| --- | --- | --- | --- | --- | --- |
| Author (published year) | Design | Patients | Intervention (I) and control (C) | Number of patients | Δ serum albumin level |
| Smart HL, et al. (1990) ^38^ | RCT | Ascites from  liver cirrhosis | I: CART | 20 | +0.1 |
|  |  |  | C: paracentesis with ALBP infusion  (human albumin: 40 g regardless of removed ascites volume) | 20 | +0.7 |
| Bruno S, et al. (1992) ^39^ | RCT | Ascites from  liver cirrhosis | I: CART | 17 | No data |
|  |  |  | C: paracentesis with ALBP infusion  (human albumin: 4-6 g for each liter of removed ascites) | 18 | No data |
| Graziotto A, et al. (1997) ^40^ | RCT | Ascites from  liver cirrhosis | I: CART | 12 | +0.1±0.5 |
|  |  |  | C: paracentesis with ALBP infusion  (human albumin: 6 g for each liter of removed ascites) | 12 | +0.3±0.4 |
| Zaak D, et al. (2001) ^41^ | RCT | Ascites from  liver cirrhosis | I: CART | 14 | +0.17 |
|  |  |  | C: paracentesis with ALBP infusion  (human albumin: 5-8 g for each liter of removed ascites) | 21 | +0.25 |

Δ serum albumin level (before and after drainage treatment) is represented as the mean±SD. SD was not indicated when unknown.


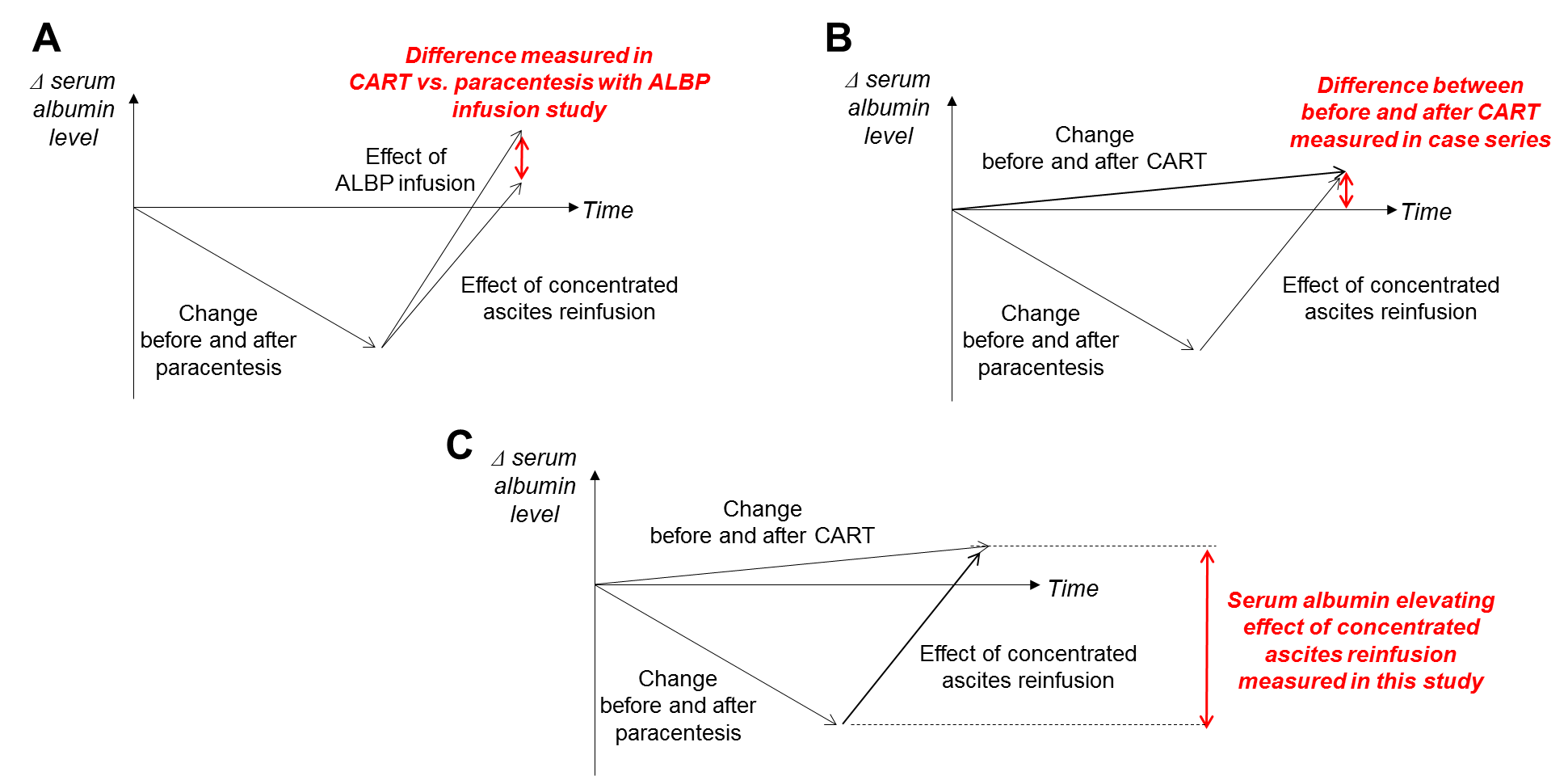


***Supplementary Figure S4. Conceptual diagram explaining the difference between this study and previous studies.*** A) Study of CART vs. paracentesis with ALBP infusion. B) Case series study on CART. C) Current study comparing CART with simple paracentesis to evaluate the effect of concentrated ascites reinfusion.


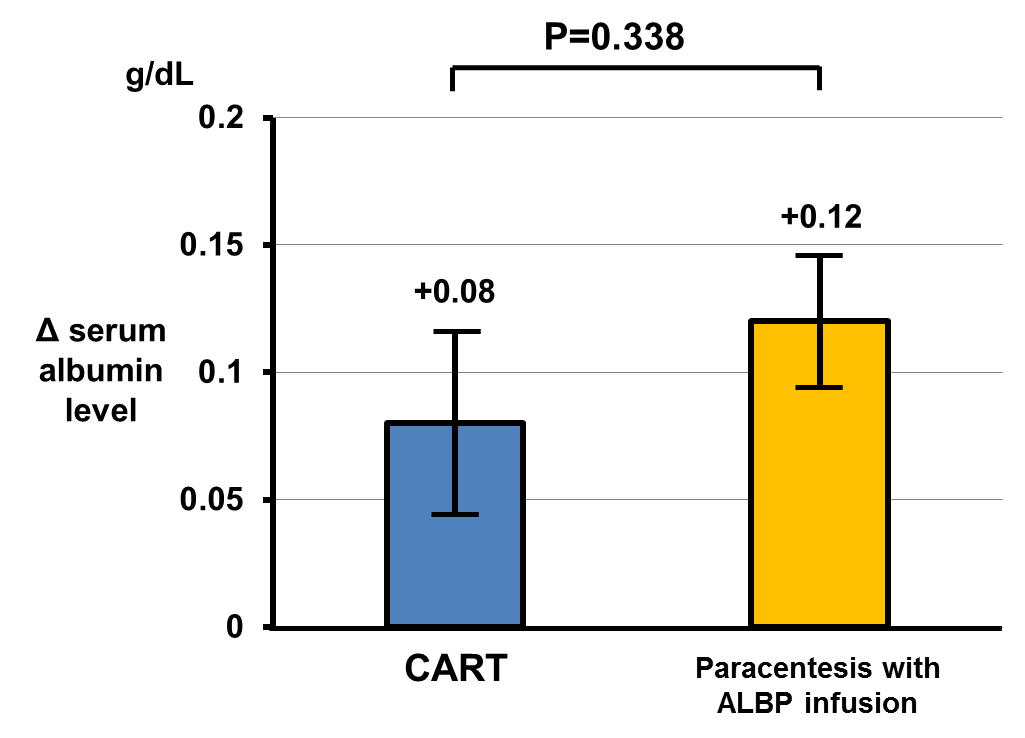


***Supplementary Figure S5. Comparison of Δ serum albumin level between CART alone and paracentesis with ALBP infusion.*** CART alone group, N=77; Paracentesis with ALBP infusion group, N=41. The Student’s *t*-test was used. Blue bar, CART alone group; yellow bar, paracentesis with ALBP infusion group. Error bars represent standard error.


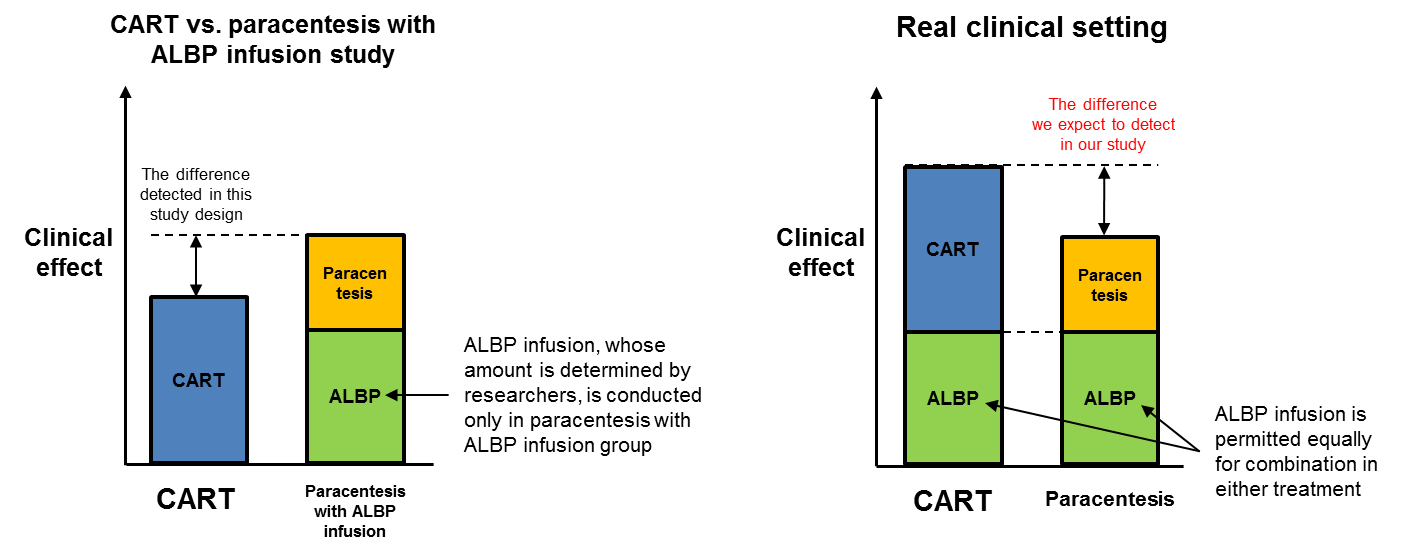


***Supplementary Figure S6. Diagram explaining the clinical importance of this study.***


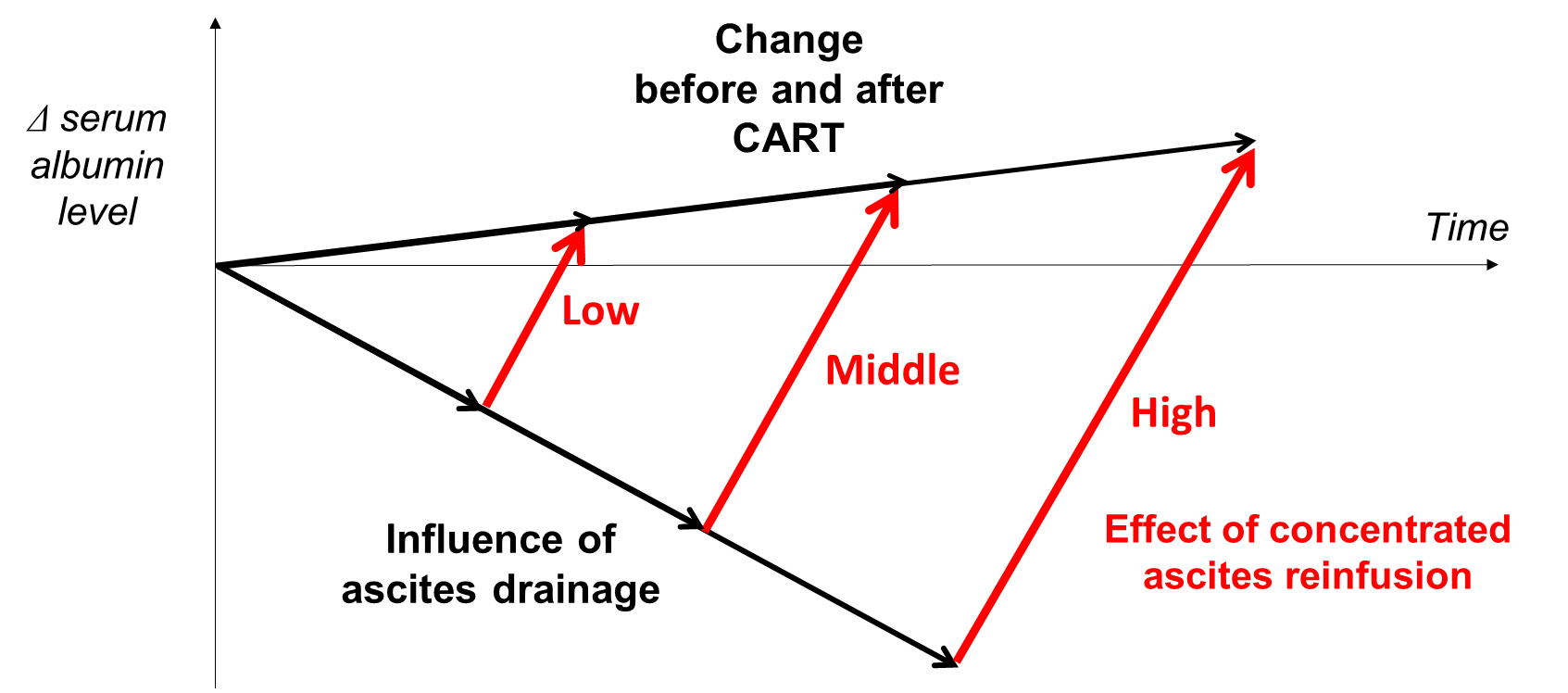


***Supplementary Figure S7. Conceptual time-course diagram representing the change in serum albumin level during CART based on the results of this study.***


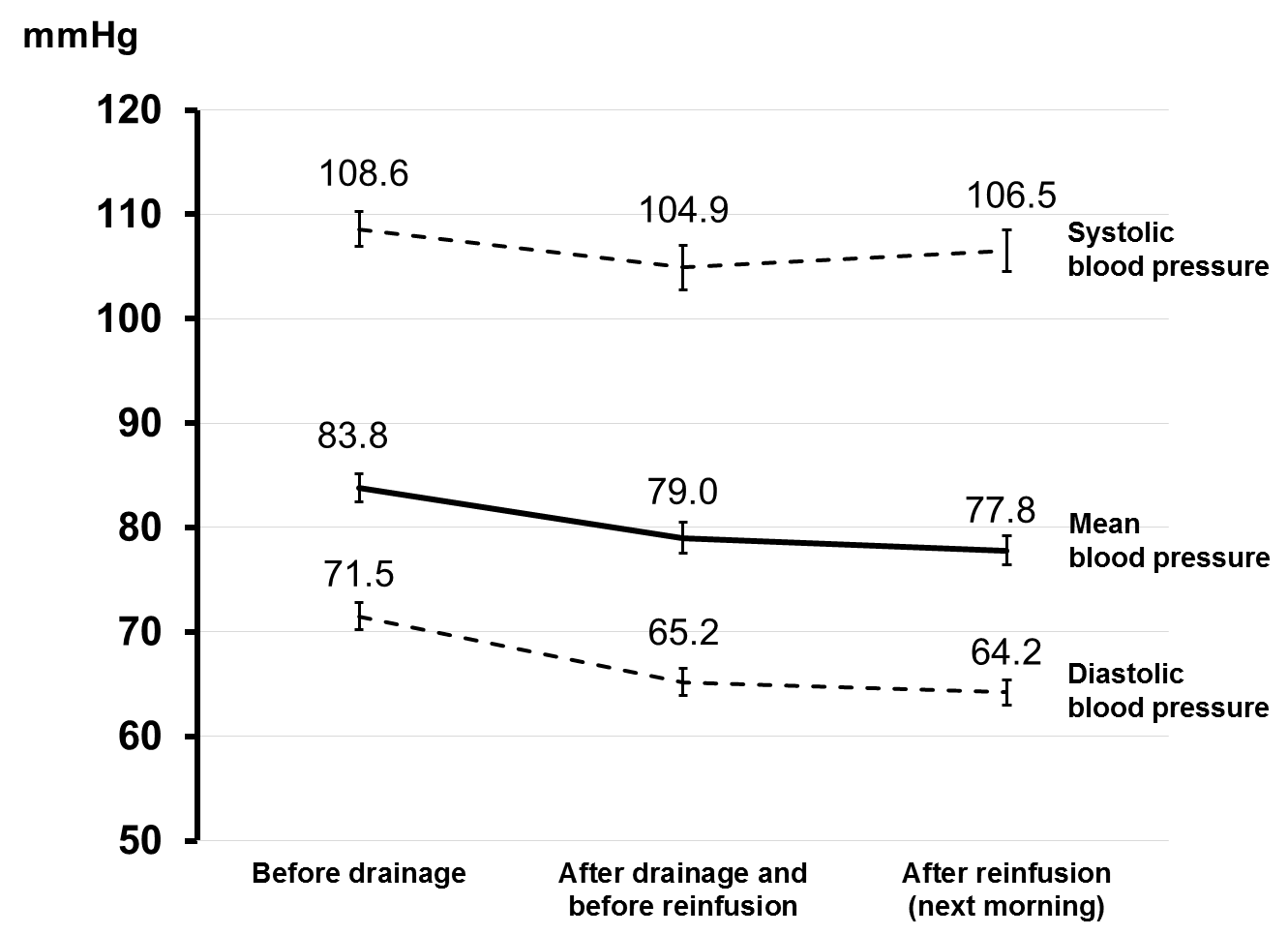


***Supplementary Figure S8. Changes in blood pressure during CART.*** Solid line, mean blood pressure; dotted lines, systolic and diastolic blood pressure. Error bars represent standard error.
